# Motor deficits seen in microglial ablation mice could be due to non-specific damage from high dose diphtheria toxin treatment

**DOI:** 10.1038/s41467-022-31562-3

**Published:** 2022-07-05

**Authors:** Jiyun Peng, Qian Zou, Min-Jie Chen, Chao-Lin Ma, Bao-Ming Li

**Affiliations:** 1 Institute of Life Science, Nanchang University, 330031 Nanchang, China; 2 Department of Physiology and Institute of Brain Science, School of Basic Medical Sciences, Hangzhou Normal University, 311121 Hangzhou, China

**Keywords:** Microglia, Cellular neuroscience

**arising from** S. Rubino et al. *Nature Communications* 10.1038/s41467-018-05929-4 (2018)

Systemic microglial cell depletion is a commonly used research strategy. A study by Rubino et al. reported that acute and synchronous microglia depletion and subsequent repopulation induce gray matter microgliosis, neuronal death in the somatosensory cortex and ataxia-like behavior^1^. This phenomenon is inconsistent with our previous observation using the same diphtheria toxin depletion strategy. Thus, we repeated the same experiments as they did and paralleled with carefully designed controls. We did see the motor deficits in the microglia depletion mice using their DT dose and injection scheme. However, the same phenomenon was also seen in WT mice that received the same DT treatment. In addition, we reduced the DT dose in another group of DTR mice, but still depleted microglia efficiently. The motor deficits were not seen in the low dose DT-treated DTR nor WT mice. Therefore, we argue that the motor deficits seen by Rubino et al. may be due to a non-specific high dose DT-induced damage but not microglial repopulation itself.

In Rubino et al.’s report^1^, the increased ataxia scores and rotarod performance decline were quite robust in microglia-depleted mice. The results were surprising to us. Since Gan et al.^2^ (JAX #021160) and Jung et al.^3^ (JAX #020940) developed CX3CR1-CreERT2 transgenic mice, this microglia ablation strategy using DT administration to microglial DTR-expressing mice or induction of DTA expression in microglia has been employed by several laboratories^2,4–8^. Rubino et al. was the first one to report a severe motor deficit after microglia ablation and subsequent repopulation.

Bruttger et al. ^8^ first described the source and cell character of the repopulated microglia after DT ablation. They claimed that “DTR^MG^ mice do not show any obvious pathological or behavioral phenotype after microglia ablation”. We also observed normal basal rotarod performance one day after DT treatment in the DTR mice. The mice looked healthy during a 2-week length of pain behavioral tests after DT treatment in our study^6^. Another pharmacological microglia depletion method, using PLX3397 or PLX5562 that targets colony-stimulating factor 1 receptor (CSF1R), also can induce microglia depletion. Microglial repopulation after drug withdrawal in that model was rapid as well. Several papers have reported long-term observation in the PLX depletion model and did not see motor deficit from acute microglia ablation to late repopulation^9,10^. Rubino et al. used PLX5562 to rescue DT-induced motor deficit in the DTR mice.

Therefore, the phenomenon they observed was quite puzzling to us. Since similar phenotype were not seen by other groups, our view was that rapid microglial repopulation was most likely not be the reason. We sought to investigate the underlying mechanism for Rubino et al.’s observations.

We noted that Rubino et al. used 3 daily doses of 1.0 μg DT intraperitoneally (i.p.), while we previously used two doses with a 48 h interval. Therefore, we speculated that the additional DT dose could be the reason for the observed effects. Thus, we did a pilot experiment with freshly ordered DT. From this, 3 daily doses of 1.5 and 2.0 μg were i.p. injected to naïve WT mice (4 and 2 mice, respectively). Surprisingly, all the mice got heavily sick within a week and eventually died at day 9–12 after the injection. Therefore, 3 doses of 1.0 μg DT could be potentially harmful to even WT mice. It is possible that the observed phenomenon in Rubino et al.’s study was due to unexpected DT side effects that were unrelated to microglia ablation or repopulation.

To test this potential caveat, we repeated the same experiments using both DTR and WT mice with different doses of DT. In the first group, we used the same dose and injection paradigm as that in the Rubino et al. study. Three doses of DT (1.0 μg in 0.2 ml PBS) were i.p. injected daily. In the second group, we used the low dose DT paradigm with two doses of DT (0.5 μg in 0.2 ml PBS) i.p. injected at a 48 h interval. We performed behaviors and immunostaining analysis on the mice treated with high and low dose of DT. To this end, ataxia score and body weight were measured from day 4 to day 10 after the last DT injection. Accelerating rotarod (0–60 RPM in 5 min) was tested on the last day. In the end, the mice were sacrificed for Iba1 staining at day 10.

Similar to Rubino et al.’s report, we observed ataxia-like behaviors in the DTR mice with high dose DT treatment. However, the WT control mice that received the same dose of DT also displayed similar body weight loss, ataxia-like behavior and rotarod performance decline (Fig. 1a–c). Next, we analyzed the activation of repopulated microglia 10 days after DT treatment (Fig. 2a). Compared with low dose of DT treatment, microglial activation was observed after high dose of DT treatment in both WT and DTR mice (Fig. 2b–d). Iba1 staining showed that microglia had increased cell density (Fig. 2b), ameboid-like morphology with enlarged cell body (Fig. 2c) and retracted processes in the cortex of both WT and DTR mice after high dose DT treatment. Detailed sholl analysis of individual microglia found that the interactions at 16 μm and farther from the soma were significantly reduced in the high dose DT-treated WT and DTR mice compared to no DT-treated WT control and low dose DT-treated WT mice. The farthest distance that processes reached was reduced from 40.47 ± 1.40 to 28.12 ± 1.33 and 26.38 ± 1.05 μm, respectively. One-way ANOVA test revealed no sholl interaction difference between the high dose DT-treated WT and DTR mice. To further investigate the time course of microglia activation after high dose DT treatment, we obtained brain slices on day 4 and 7 from high dose DT-treated WT mice and performed Iba1 staining. The microglial cell densities gradually increased from day 4 to day 10. Ameboid-like microglia were seen on day 7 samples, but not yet on day 4 samples (Fig. S1). Therefore, high dose DT-induced non-specific microglia activation takes a much longer time than the specific cell depletion time in DTR mice. Together, our results indicated that high dose DT treatment non-specifically caused ataxia-like behavior and microglia activation even in WT mice.

**Fig. 1:**
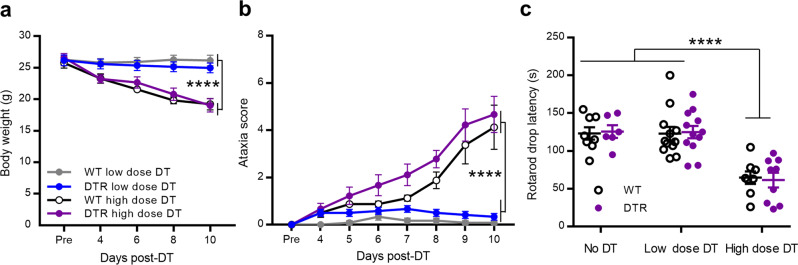
High dose diphtheria toxin non-specifically induced motor deficits. **a** Both WT (*n* = 8) and CX3CR1^CreER/+^/R26^iDTR/+^ (DTR) (*n* = 9) mice showed similar and significant body weight decrease after high dose DT (1.0 μg × 3) treatment. While both groups of low dose DT (0.5 μg×2) (*n* = 12 for each group) treated mice maintained stable body weight within two weeks. (two-way ANOVA with repeated measurement, *F* (3, 37) = 7.763, ***p = 0.0004 for group effects. *p* = 0.5985 between WT and DTR high dose groups, *p* = 0.5465 between WT and DTR low dose groups.). **b** Ataxia score assessment showed significant progressive ataxia behavior in both WT and DTR mice with high dose DT treatment; In the low dose DT treated groups, 5/12 WT and 10/12 DTR mice transiently showed slight ledge walking problem (score 1), and most of the mice fully recovered in the last observation (score 0, 4/5 WT, and 8/10 DTR mice); other low dose DT-treated mice remained normal (score 0) all the time. Mouse numbers were equal to panel **a** for each group (two-way ANOVA with repeated measurement, *F* (3, 37) = 22.74, ****p < 0.0001, for overall group effects. *p* = 0.2425 between WT and DTR high dose groups, *p* = 0.0025 between WT and DTR low dose groups and *p* > 0.3 for day 8 and later with post-hoc Sidak’s test, two sides.). **c** Rotarod test showed significant shorter drop latency for both high dose DT-treated WT and DTR mice comparing to no DT-treated controls (*n* = 8 for WT control, *n* = 6 for DTR control). While both low dose DT-treated groups showed similar rotarod performance as controls (****p < 0.0001, un-paired *t*-test, two sides). All the DT-treated mouse were used for data collection of (**a**–**c**). Data were presented as mean ± SEM. Source data are provided as a Source Data file for (**a**–**c**).

**Fig. 2:**
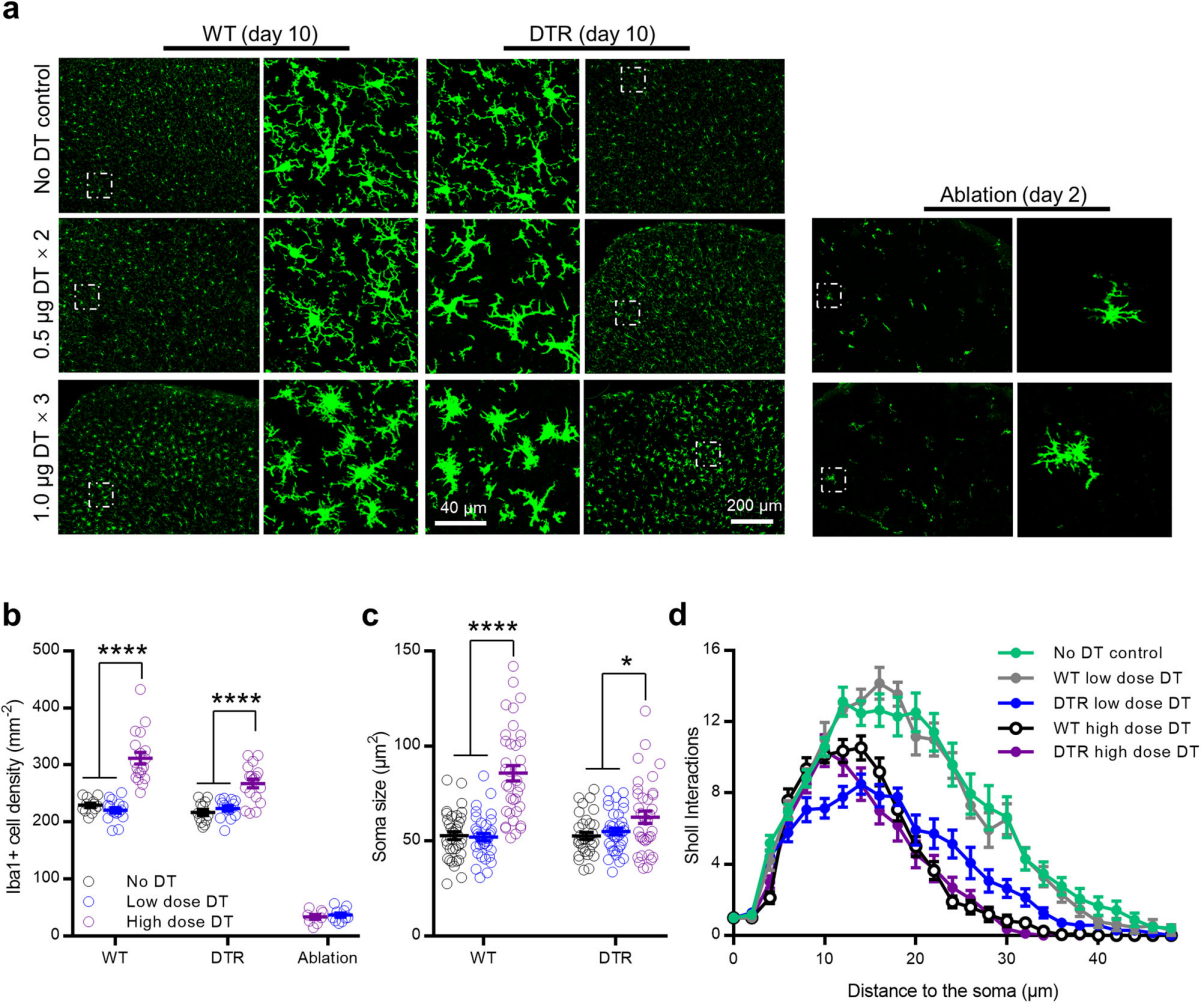
High dose diphtheria toxin non-specifically induced microglia activation. **a** Representative Iba1 staining images from cortex. The enlarged images were from the areas indicated by the square frame of each group. **b** Cortical Iba1+ microglial cell densities showed that low dose DT (*n* = 16) treatment did not alter microglia density in the WT mice, but high dose (*n* = 19) significantly increased the density compared with no DT WT control (*n* = 12); Repopulated microglia in the low dose DT (*n* = 14) treated DTR mice reached basal cell density (*n* = 12) at 10 days, but was significantly increased in the high dose DT (*n* = 18) group. The microglia depletion efficiencies between the high (*n* = 8) and lose dose (*n* = 10) DT groups were comparable. (****p < 0.0001, *t*-test, two sides). **c** Soma size measurement showed dramatic cell body enlargement in the high dose (n = 36) but not low dose DT (*n* = 36) treated WT mice, and in the high dose (*n* = 36) but not low dose DT (*n* = 36) treated DTR mice (*n* = 36 for no DT WT, *n* = 33 for no DT DTR group, ****p < 0.0001, *p = 0.0129, *t*-test, two sides). **d** Sholl analysis of individual Iba1+ microglia cells showed that the low dose DT (*n* = 13) treatment in WT mice did not alter microglia morphology compared with no DT controls (*n* = 17, WT and DTR control data were pooled together, *p* = 0.9837). Microglia from both high dose DT treated WT (*n* = 17) and DTR (*n* = 16) mice showed similar less interactions than no DT controls (*p* < 0.0001). Microglia from the low dose DT treated DTR (*n* = 14) mice also showed less interactions than no DT controls (*p* < 0.0001) Two-way ANOVA with re*p*eated measurement (**b**–**d**, images collected from 3 mice for each group, except *n* = 5 mice for the low dose DT ablation at 2 days). Data were presented as mean ± SEM. Source data are provided as a Source Data file for (**b**–**d**).

With low dose DT treatment, both WT and DTR mice appeared normal with body weight maintenance and rotarod performance on the final test day. Some of the mice (5/12 WT and 10/12 DTR) transiently showed a slight ledge walking problem (score 1, other mice were score 0 all the time), but most of them recovered on the last observation day (score 0, 4/5 WT and 8/10 DTR mice). Iba1 staining showed normal microglia cell density and morphology (indicated by soma size and sholl analysis) in the low dose DT-treated WT mice. In the low dose DT-treated DTR mice, the repopulated microglia cell density was comparable to the no DT control mice. The microglia morphology in the DTR mice did not return to normal yet with less ramified processes (indicated by reduced sholl interactions). However, the soma size was similar to the no DT control and the farthest distance the processes reached (36.71 ± 1.49 μm) was close to the no DT control (40.47 ± 1.40 μm) as well (*p* = 0.077, *t*-test, two sides). This phenomenon was consistent with the previous observation in the spinal cord after DT treatment in DTR mice^6^, or the repopulated microglia with CSF1R inhibitor treatment^10^. To confirm whether this low dose DT could ablate microglia efficiently in the DTR mice, we examined Iba1 staining at 48 h after the last DT injection for both the high dose and low dose DT-treated DTR mice. We found that the ablation efficiencies were similar between the high dose and low dose groups (Fig. 2a, b, 85.4 ± 1.5% and 84.0 ± 1.7% microglial ablation, data obtained from 3 and 5 mice, respectively). Thus, the low dose DT treatment was sufficient to induce microglial ablation in the DTR mice, but relatively milder activation of repopulated microglia.

Taking our present and previous data^6^ together, we believe that DT-induced microglia ablation and subsequent repopulation itself could not cause ataxia-like behaviors and motor deficits in the DTR mice. Therefore, in our view the motor deficit observed by Rubino et al. may be due to a high dose DT-treatment regimen which induced non-specific brain cell damage.

Systemic i.p. injection is a common delivery method for in vivo DT treatment. The DT dose and the injection scheme varied in the literature for different cell targets. For the CX3CR1^CreER/+^/R26^iDTR/+^ mice, the dose was different between Parkhurst et al. (1 μg per injection, 3 daily injections)^2^ and Bruttger et al. (0.5 μg per injection, 3 daily injections)^8^ as well. In the original method paper describing the iDTR mice, 100 ng per injection was used^11^. To our knowledge, all of those previous studies included DT-treated control mice that did not express DTR. No side effects were described in the control mice in those reports. In the Rubino et al. study, the control setting in our view was not described sufficiently clearly. For a detailed comparison, Table S1 lists the methods and microglia depletion efficiencies in recent studies using DTR/DTA mice. The factors that determined the DT dose choice should include the DT product source and the stock solution preparation. The DT efficiency could vary from different batches of the product. In addition, in our experience, the stock time and condition before using could affect the drug efficiency. DT-induced apoptotic cell death is very sensitive and efficient. A single molecule of active DT-A subunit is sufficient to kill a eukaryotic cell^12^, because DT-A is quite stable in the intra-cellular environment with the present of NAD^13^. Although the DT receptor is not expressed in naïve mouse cells, there could be chance for the molecule to enter into a cell via other unknown mechanism and kill it in a long time. That could potentially explain why non-specific neuronal cell death and motor deficit took much longer time to happen than the DTR-mediated microglia depletion. High dose of DT exposure could potentially increase the risk of non-specific cell damage. Therefore, a control group of mice receiving the same dose of DT is critical for all experiments using DT ablation approaches. Experimenters should be aware of possible side effects when using DT on mice.

## Methods

### Animals

All experimental procedures were approved by the Institutional Animal Care and Use Committee of Nanchang University. We followed the guidelines set forth by the Guide of the Care and Use of Laboratory Animals 8th Edition. CX3CR1-CreER (#021160) mice and ROSA26-iDTR (#007900) mice were origin from the Jackson laboratory. Wild type C57BL6/N mice were obtained from SLAC Laboratory Animal Co. Ltd. Only male mice were used for the whole study. The genotypes were blinded to the experimenters. Mice were group (4–5 per cage) housed in 12/12 light/dark cycle, 23 ± 1 °C vivarium environment. Food and water were available *ad libitum*.

Tamoxifen (150 mg/kg, 20 mg/ml in corn oil, 4 i.p. injections with 48 h intervals; Sigma) was injected into 6–8 weeks old CX3CR1^CreER/+^/R26^iDTR/+^ mice to induce DT receptor expression in microglia. DT injection was done at 3 weeks after the last tamoxifen treatment.

### DT administration

New DT (Merck Millipore, #322326) was prepared as a 100 μg/ml stock solution in PBS, separated into tubes of 100 μl volume and stored at −20 °C. The stock solution was diluted to 5 μg/ml for the high dose injection and 2.5 μg/ml for the low dose injection. All DT injections were done within 2 weeks after the stock preparation. Fresh diluted solution was used immediately, and the remaining was discarded.

### Behavioral assessment

Ataxia scoring was assessed according to a previous published protocol that was used by the criticized paper as well^14^. Ledge test monitored the mice walking along the cage ledge; Hindlimb clasping monitored the hindlimb posture when the mice were held by the tail. Gait assessed the hindlimb walking performance. Kyphosis assessed the dorsal curvature of the spine. Each individual test was scored on a scale of 0–3 with 0 representing normal and 3 being the most severe manifestation. All 4 scores were added together as the final score. Each test was performed at least 3 times.

Rotarod tests were performed on an accelerated Rotarod (30 mm in diameter, www.softmaze.com). The mice were first placed on the rotarod with 5 RPM speed for 5 min habituation. For the test, the speed started from 0 RPM, and accelerated to 60 RPM in 300 s. The time latency that mice fell from the rotarod was recorded.

### Fluorescent immunostaining

Mice were deeply anaesthetized with isoflurane (5% in O_2_) and perfused transcardially with 20 ml PBS followed by 20 ml of cold 4% paraformaldehyde (PFA) in PBS. The whole brain was removed and post-fixed with the same 4% PFA for 4–6 h at 4 °C. The samples were then transferred to 30% sucrose in PBS for at least 48 h in dark conditions. Sample sections (18 μm in thickness) were prepared on gelatin-coated glass slide with a cryostat (Leica). The sections were blocked with 5% goat serum and 0.3% Triton X-100 (Sigma) in TBS buffer for 45 min, and then incubated overnight at 4 °C with a primary antibody for rabbit-anti-Iba1 (1:800, Abcam, Catalog ab178846). The sections were then incubated for 90 min at room temperature, with secondary antibodies (1:500, Alexa Fluor 546, Life Technologies, Catalog A-21085). The sections were mounted with Fluoroshield™ with DAPI (Sigma). Fluorescent images were obtained with a confocal microscope (Olympus). Cell counting, soma size and sholl analysis were quantified using the ImageJ software (ImageJ 1.52a, National Institutes of Health, Bethesda, MD).

### Statistical analysis

Data were presented as mean ± SEM. Student’s *t*-test and ANOVA test were performed using GraphPad (GraphPad Prism 7, GraphPad software Inc.) to establish significance. *p < 0.05, **p < 0.01, ***p < 0.001.

### Reporting summary

Further information on research design is available in the Nature Research Reporting Summary linked to this article.

## Supplementary information

Supplementary Information

Reporting Summary

## Data Availability

All raw data for the statistic figures are available in the source data file. All relevant image data and analysis are available on request from the corresponding author. Source data are provided with this paper.

## Source data

Source Data
