# Supplementary material for: Motor deficits seen in microglial ablation mice could be due to non-specific damage from high dose diphtheria toxin treatment
Source: Nat Commun. 2022 Jul 5;13:3874. doi: 10.1038/s41467-022-31562-3 (PMC9256727; doi:10.1038/s41467-022-31562-3)
Supplement: Supplementary file 1 — Supplementary Information [file 41467_2022_31562_MOESM1_ESM.pdf]

Supplementary Information

Motor deficits seen in microglial ablation mice could be due to non-specific damage from high dose diphtheria toxin treatment

Jiyun Peng[1*], Qian Zou[1], Min-Jie Chen[1], Chao-Lin Ma[1], Bao-Ming Li[1,2],

[1]Institute of Life Science, Nanchang University, Nanchang, 330031, China,

[2] Department of Psychology and Institute of Brain Science, School of Basic Medical Sciences, Hangzhou Normal University, Hangzhou, 311121, China

[*]Correspondence should be addressed to: J.P. (pengjy@ncu.edu.cn)

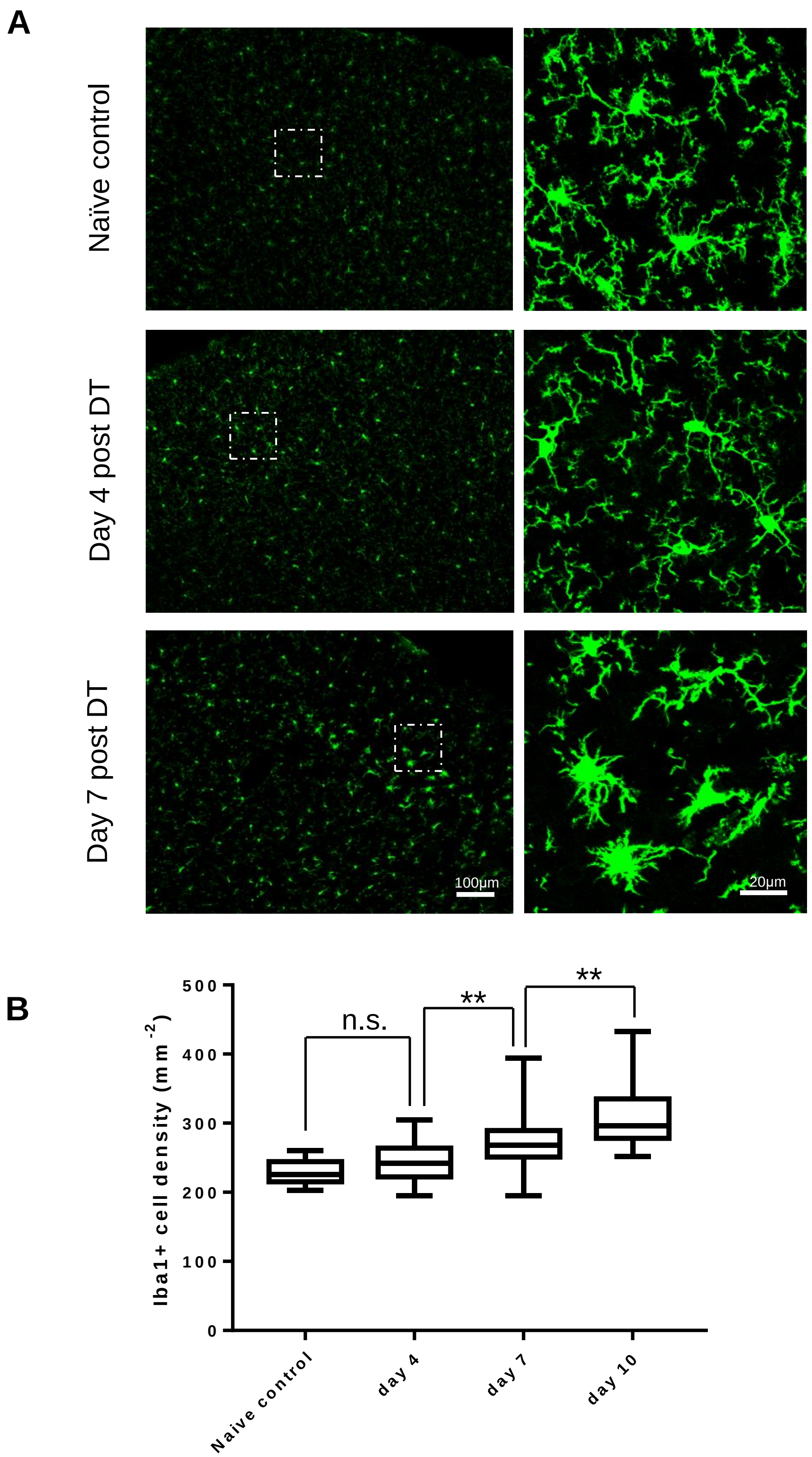

Supplementary figure 1: Microglia were gradually activated in the WT cortex from day 4-10 after DT administration. A) Representative Iba1 staining images showing activated microglia at day 7, but not day 4 after high dose DT treatment in WT mice. Left, 10X images; Right, 40X images from the areas indicated by the square frames in the left. **B)** Statistic data showing microglia cell densities in the cortex from different time point samples (N = 22 slices for Naïve control, 20 for day 4, 42 for day 7 and 19 for day 10 group). Data were presented as box and whiskers (min, max). One ANOVA analysis indicated significant time effects among groups ($F(3, 99)= 22.25$, $p < 0.0001$).Multiple comparisons with Turkey's test revealed significant differences between day 4 and day 7($p = 0.0084$), and between day 7 and day 10 ($p = 0.0011$). n.s., non-significant. Images were collected from 3 mice for each group, except N = 5 for day 10 group.

Source	DT dose	Tamoxifen dose	Measurement method	mice model & age	microglia depletion efficiency
Parkhurst et. al*., Cell, 2013*	1 µg * 3, i.p. with 24 hr interval	50 µg * 3 per intragastric injection on P1-3	CX3CR1-EYFP flow cytometry	CX3CR1-CreER/iDTR, P30	99% for CNS
Bruttger et. al., *Immunity, 2015*	0.5 µg * 3, i.p. with 24 hr interval	2 mg per s.c. injection on P12 and P14	CD11b flow cytometry & Iba1 immunostaining	CX3CR1-CreER/iDTR, 8 weeks	80% for cortex
Wang et. al., *J. Neurosci. 2016*	/	500 mg/kg, oral gavage with 2 and 5 day intervals in adults	Iba1 immunostaining	CX3CR1-CreER/iDTA, 2-3 months	99.6% for retina
Peng et. al., *Nat. Commun. 2016*	1 µg * 2, i.p. with 48 hr interval	150 mg/kg * 4, i.p. with 48 hr interval in adults	Iba1 immunostaining	CX3CR1-CreER/iDTR, 7-9 weeks	94% for spinal cord, 85% for cortex
Rubino et. al*., Nat. Commun. 2018*	1 µg * 3, i.p. with 24 hr interval	10 µg * 5, i.p. with 24 hr interval	CX3CR1-EYFP flow cytometry	CX3CR1-CreER/iDTR,	95% for brain

Supplementary Table 1: Available published Diphtheria Toxin induced microglia ablation methods and the depletion efficiencies.
