# Supplementary material for: Motor deficits seen in microglial ablation mice could be due to non-specific damage from high dose diphtheria toxin treatment
Source: Nat Commun. 2022 Jul 5;13:3874. doi: 10.1038/s41467-022-31562-3 (PMC9256727; doi:10.1038/s41467-022-31562-3)
Supplement: Supplementary file 2 — Reporting Summary [file 41467_2022_31562_MOESM2_ESM.pdf]

nature portfolio

Corresponding author(s): Jiyun Peng

Last updated by author(s): Mar 12, 2022

Reporting Summary

Nature Portfolio wishes to improve the reproducibility of the work that we publish. This form provides structure for consistency and transparency in reporting. For further information on Nature Portfolio policies, see our Editorial Policies and the Editorial Policy Checklist.

Statistics

For all statistical analyses, confirm that the following items are present in the figure legend, table legend, main text, or Methods section.

n/a	Confirmed	
☐	☒	The exact sample size (n) for each experimental group/condition, given as a discrete number and unit of measurement
☐	☒	A statement on whether measurements were taken from distinct samples or whether the same sample was measured repeatedly
☐	☒	The statistical test(s) used AND whether they are one- or two-sided<br>*Only common tests should be described solely by name; describe more complex techniques in the Methods section.*
☐	☒	A description of all covariates tested
☐	☒	A description of any assumptions or corrections, such as tests of normality and adjustment for multiple comparisons
☐	☒	A full description of the statistical parameters including central tendency (e.g. means) or other basic estimates (e.g. regression coefficient) AND variation (e.g. standard deviation) or associated estimates of uncertainty (e.g. confidence intervals)
☐	☒	For null hypothesis testing, the test statistic (e.g. F, t, r) with confidence intervals, effect sizes, degrees of freedom and P value noted<br>*Give P values as exact values whenever suitable.*
☒	☐	For Bayesian analysis, information on the choice of priors and Markov chain Monte Carlo settings
☒	☐	For hierarchical and complex designs, identification of the appropriate level for tests and full reporting of outcomes
☐	☒	Estimates of effect sizes (e.g. Cohen's d, Pearson's r), indicating how they were calculated

Our web collection on statistics for biologists contains articles on many of the points above.

Software and code

Policy information about availability of computer code

Data collection	ImageJ 1.52a
Data analysis	ImageJ 1.52a, GraphPad Prism 7

For manuscripts utilizing custom algorithms or software that are central to the research but not yet described in published literature, software must be made available to editors and reviewers. We strongly encourage code deposition in a community repository (e.g. GitHub). See the Nature Portfolio guidelines for submitting code & software for further information.

Data

Policy information about availability of data

All manuscripts must include a data availability statement. This statement should provide the following information, where applicable:

- Accession codes, unique identifiers, or web links for publicly available datasets
- A description of any restrictions on data availability
- For clinical datasets or third party data, please ensure that the statement adheres to our policy

All individual data for the statistic figures are available in the uploaded source data file. All relevant image data and analysis are available on request from the corresponding author.

Field-specific reporting

Please select the one below that is the best fit for your research. If you are not sure, read the appropriate sections before making your selection.

☒ Life sciences ☐ Behavioural & social sciences ☐ Ecological, evolutionary & environmental sciences

For a reference copy of the document with all sections, see nature.com/documents/nr-reporting-summary-flat.pdf

Life sciences study design

All studies must disclose on these points even when the disclosure is negative.

Sample size	For animal studies, sample sizes were selected based on previous mouse study literatures. For image studies, all available collections from the mouse samples were used for statistic analysis.
Data exclusions	No data were excluded.
Replication	The major experiments were repeated in two sets using two batches of freshly ordered DT. Each set of animal experiment was repeated with multiple mice. Each imaging experiment was repeated with samples from at least 3 mice.
Randomization	litter mate mice were randomly assigned to low dose DT and high dose DT groups and blind to the experimenter.
Blinding	Experimenters were blind to the group allocation of each mouse until all behavioral and image data collection, and individual image data analysis were completed.

Reporting for specific materials, systems and methods

We require information from authors about some types of materials, experimental systems and methods used in many studies. Here, indicate whether each material, system or method listed is relevant to your study. If you are not sure if a list item applies to your research, read the appropriate section before selecting a response.

Materials & experimental systems

n/a	Involved in the study	
☐	☒	Antibodies
☒	☐	Eukaryotic cell lines
☒	☐	Palaeontology and archaeology
☐	☒	Animals and other organisms
☒	☐	Human research participants
☒	☐	Clinical data
☒	☐	Dual use research of concern

Methods

n/a	Involved in the study	
☒	☐	ChIP-seq
☒	☐	Flow cytometry
☒	☐	MRI-based neuroimaging

Antibodies

Antibodies used	rabbit-anti-Iba1 (Abcam, Catalogue ab178846), Rabbit anti-Goat Alexa Fluor 546 (Life Technologies, Catalogue A-21085)
Validation	Iba1 (Suitable for: Flow Cyt (Intra), IHC-P, WB, ICC/IF; Reacts with: Mouse, Rat, Human)

Animals and other organisms

Policy information about studies involving animals; ARRIVE guidelines recommended for reporting animal research

Laboratory animals	C57BL6/N (SLAC laboratory animal CO. LTD, Changsha, Hunan Province, China), CX3CR1-CreER (jax #021160) and ROSA26-iDTR (jax #007900) transgenic mice with C57BL6 background male mice that aged from 6-12 weeks were used.
Wild animals	This study did not involve wild animals.
Field-collected samples	This study did not involve samples collected from field.
Ethics oversight	All experimental procedures were approved by the Institutional Animal Care and Use Committee of Nanchang University.

Note that full information on the approval of the study protocol must also be provided in the manuscript.
